# Real-Time Forecasting of Hand-Foot-and-Mouth Disease Outbreaks using the Integrating Compartment Model and Assimilation Filtering

**DOI:** 10.1038/s41598-019-38930-y

**Published:** 2019-02-25

**Authors:** Zhicheng Zhan, Weihua Dong, Yongmei Lu, Peng Yang, Quanyi Wang, Peng Jia

**Affiliations:** 1 State Key Laboratory of Remote Sensing Science, Beijing Key Laboratory for Remote Sensing of Environment and Digital Cities, Research Centre of Geospatial Cognition and Visual Analytics, and Faculty of Geographical Science, Beijing Normal University, Beijing, 100875 China; 2 Department of Geography, Texas State University, San Marcos, TX 78666-4684 USA; 3 Institute for Infectious Disease and Endemic Disease Control, Beijing Centre for Disease Prevention and Control, Beijing, 100013 China; 4 Faculty of Geo-Information Science and Earth Observation (ITC), University of Twente, Enschede, 7500 The Netherlands; 5 International Initiative on Spatial Lifecourse Epidemiology (ISLE), Enschede, The Netherlands

## Abstract

Hand-foot-and-mouth disease (HFMD) is a highly contagious viral infection, and real-time predicting of HFMD outbreaks will facilitate the timely implementation of appropriate control measures. By integrating a susceptible-exposed-infectious-recovered (SEIR) model and an ensemble Kalman filter (EnKF) assimilation method, we developed an integrated compartment model and assimilation filtering forecast model for real-time forecasting of HFMD. When applied to HFMD outbreak data collected for 2008–11 in Beijing, China, our model successfully predicted the peak week of an outbreak three weeks before the actual arrival of the peak, with a predicted maximum infection rate of 85% or greater than the observed rate. Moreover, dominant virus types enterovirus 71 (EV-71) and coxsackievirus A16 (CV-A16) may account for the different patterns of HFMD transmission and recovery observed. The results of this study can be used to inform agencies responsible for public health management of tailored strategies for disease control efforts during HFMD outbreak seasons.

## Introduction

Hand-foot-and-mouth disease (HFMD) is an infectious disease caused by enteroviruses. Coxsackievirus A16 (CV-A16) and enterovirus 71 (EV-71) are the two viruses responsible for most HFMD cases^1^; EV-71 tends to cause more severe and fatal cases, whereas CV-A16 has a milder outcome. HFMD mostly affects young children under the age of 10 and is characterized by symptoms of fever and vesicular sores with blisters on palms of the hands, soles of the feet, and buttocks. Moreover, HFMD has caused death in some serious cases^2^. HFMD is transmitted from person to person through direct contact with the saliva, faeces, or vesicular fluid of an infected person; it can also be transmitted indirectly through contact with contaminated items^3^. HFMD is commonly detected in areas along the west of the Pacific Ocean during spring, summer and fall. For example, multiple outbreaks have occurred in countries such as China^4^, Singapore^5^, and Japan^6^ since the 1990s but have also occurred in western countries such as Germany^7^ and Spain^8^. During 2008–2014, more than 1 million HFMD cases each year were reported in China^9^. An effective treatment for HFMD is not available^10^. Therefore, a good understanding of the distribution and transmission of HFMD and an accurate real-time forecasting model of HFMD outbreaks are critically needed for the timely control and prevention of HFMD.

Although previous studies have examined the factors influencing HFMD^11–16^, the characteristics and transmission patterns of HFMD vary across different regions and seasons, and thus the prediction of HFMD outbreaks remains a daunting task. Compartment models such as susceptible-infected-recovered (SIR) model and susceptible-exposed-infectious-recovered (SEIR) are two typical dynamic models that attempt to reflect changes in real world or simulation environment and take into account that the model components are constantly changing as a result of previous conditions and current influences^17^. These models are commonly used to predict infectious diseases^18,19^ and have been used to simulate the dynamics of the HFMD outbreak^20–24^. However, the traditional compartment model relies on a set of static conditions and model parameters that are difficult to estimate for forecasting the HFMD outbreak due to the interactions of many uncertain factors, such as weather conditions and measures to control for social interaction. Moreover, conditions contributing to an HFMD outbreak are usually dynamic, which rarely meets the presumption of a traditional compartment model. Adding to the complexity, although one virus may predominate, multiple viruses can co-contribute to HFMD outbreaks. Furthermore, previous HFMD outbreaks in China have shown two peaks during a single year^25–27^, and predicting the timing and magnitude of the second peak tends to be more complex.

Recent literature has described a new approach for forecasting infectious diseases by integrating dynamic models and the assimilation technology, which has been used for performing dynamic and real-time adjustments of a forecasting model^28,29^. In this case, preliminary predictions from a compartment model are dynamically adjusted by incorporating real-time observations through the assimilation filter to improve model prediction. This Integrated Compartment Model Assimilation Filtering approach has been used in recent years to forecast outbreaks of several infectious diseases, such as influenza^30–32^, Ebola^33^ and West Nile virus^34^. However, the prediction of HFMD outbreaks has not been yet benefited from this innovative approach.

This study used the Integrated Compartment Model and Assimilation Filtering model for real-time forecasting of HFMD outbreaks in Beijing using reported HFMD cases during 2008–2011. In particular, we (1) simulated disease occurrences over time, (2) estimated model parameters by incorporating real-time observation data, and (3) evaluated the model by comparing the weekly forecasts of HFMD to the reported data. Moreover, we examined potential associations between the dominant virus type responsible for the outbreak and the transmission pattern of HFMD, which are used as additional information for forecasting HFMD outbreaks. This approach of real-time HFMD forecasting will potentially help researchers design effective interventions for the control and prevention of HFMD.

## Results

### Descriptive Statistics

**Figure 1:**
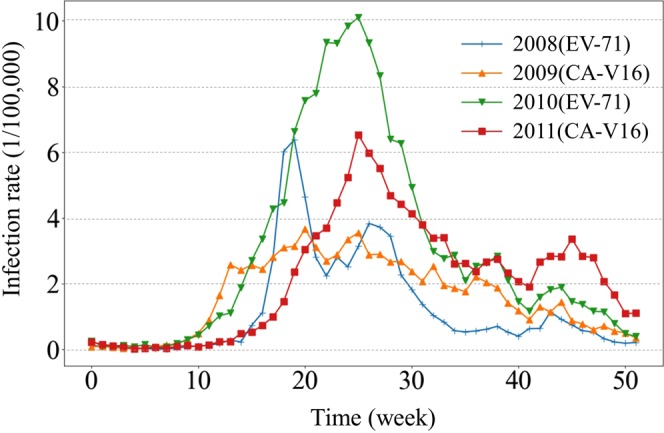
The weekly rate of HFMD infections in Beijing from 2008–11.

The weekly rate of HFMD infections in Beijing between January 1, 2008 and December 31, 2011 is shown in Fig. 1. HFMD cases occurred throughout the year, but the rate was very low in the first 10 weeks, corresponding to the period from January to March. The high-incidence period occurred from May to July. The peak week in 2010 and 2011 was week 26, and the peak weeks in 2008 and 2009 were more than one month earlier than in 2010 and 2011, week 20 in 2008 and week 21 in 2009. The peak magnitude of infection varied from year to year. The highest peak magnitude occurred in 2010, followed by 2008 and 2011. A clear second peak of infection appeared in 2008 and 2011, and the time for the second peak differed across different years. The second peak week in 2008 occurred at almost the same time as the peak in 2010 and the first peak in 2011.

### Prior Forecast and Posterior Analysis

**Figure 2:**
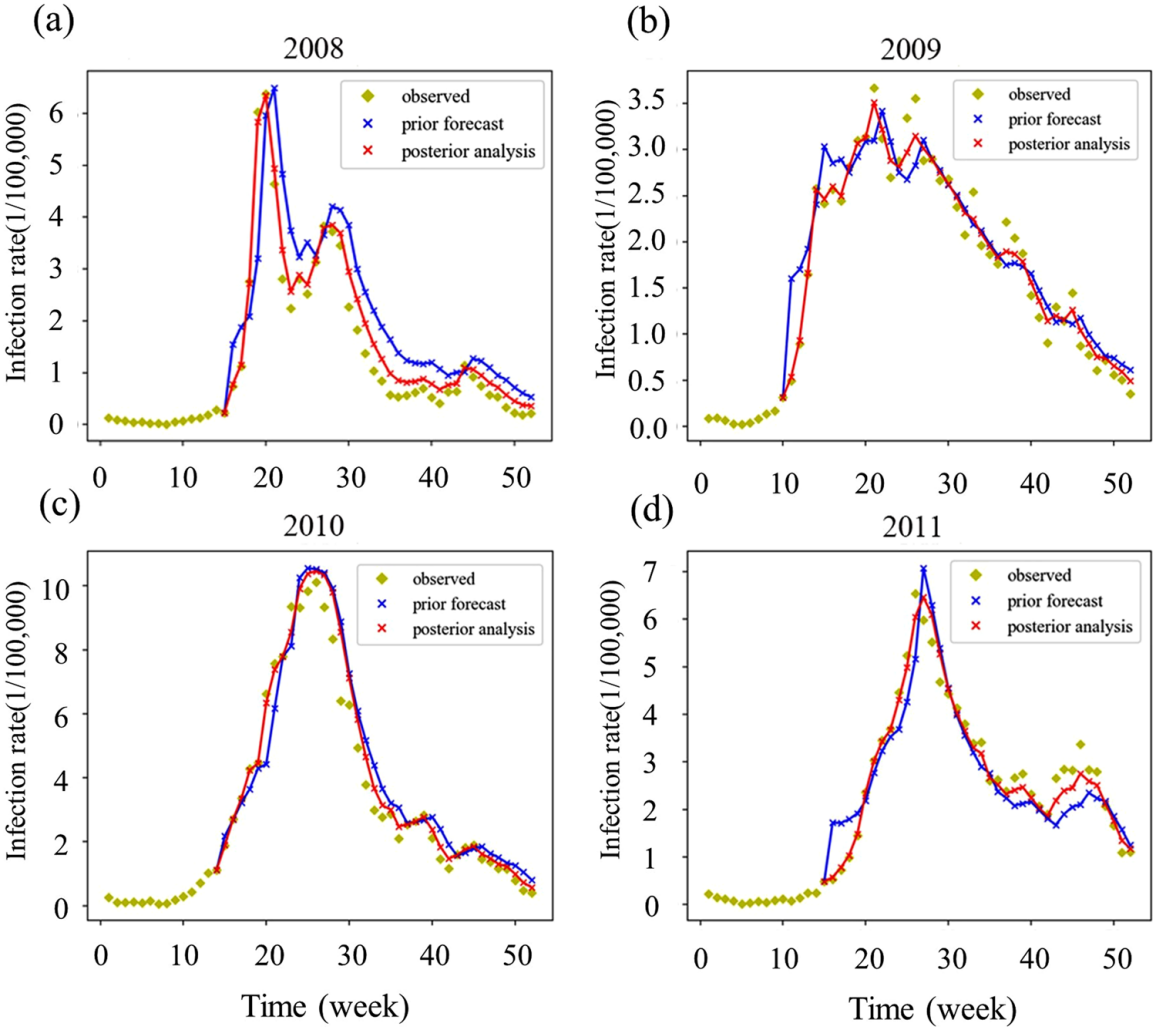
The relationships among the observed data, prior forecast outcomes, and posterior analysis outcomes in 2008 (**a**), 2009 (**b**), 2010 (**c**), and 2011 (**d**). Light green dots are the observed data for the weekly infection rate. Blue dots represent the prior forecast from the SEIR model. Red dots represent the posterior infection rate predicted using EnKF.

Prior forecasting employs the SEIR model and the current week’s variable measurements to forecast the infection rate of the next week, while the posterior analysis produces assimilation results by adjusting prior forecasts for observed data. The graphs in Fig. 2 show the observation data, prior forecast, and posterior analysis for the study period using our model. The posterior analysis successfully captured the trend of infection rates in all four years, including the second peak during the 2008 HFMD outbreak. Moreover, the results of the posterior analysis appeared to be less impacted by the outliers in the observation data. We conducted regression analyses between the observed data and the data predicted from both the prior forecast and the posterior analysis to further evaluate the difference between the results obtained from the posterior analysis and the prior forecast (see Supplementary Fig. 1); the results of this analysis also showed a better fit between the observed data and posterior analyses.

### Accuracy Assessment

**Figure 3:**
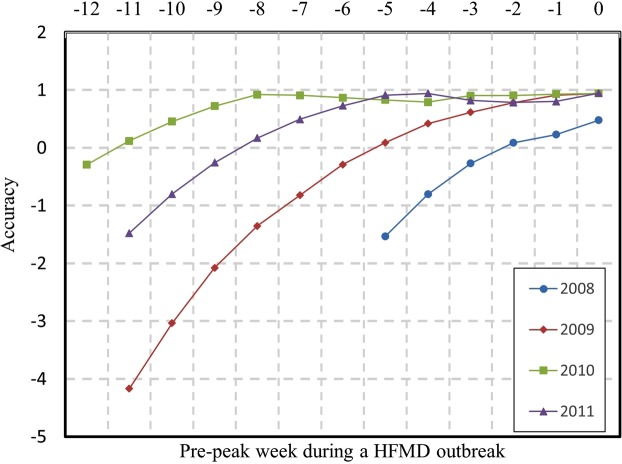
The forecast accuracy of the HFMD peak magnitudes from 2008–2011. Negative numbers on the horizontal axis represent numbers of weeks before the arrival of a peak week. The vertical axis measures the proximity of a forecasted peak infection rate is to the observed peak infection rate. A magnitude of 1 indicates a perfect forecast of the peak infection rate. The offset of the peak magnitude is defined as 1 − (*M*_*pre*_ − *M*_*obs*_)/*M*_*obs*_, where *M*_*obs*_ is the observed peak magnitude and *M*_*pre*_ is the predicted peak magnitude.

We performed real-time forecasting of HFMD for 2008–2011 (Supplementary Fig. 2) and assessed the accuracy of the forecast of the peak magnitude by comparing the forecast peak magnitude and the observed peak magnitude (Fig. 3). HFMD data were available for this study 12 weeks before the peak week in 2010 and 11 weeks before the peaks in 2009 and 2011, but only 5 weeks before the peak in 2008. Correspondingly, the peak week magnitude forecast was the most accurate for 2010 and least accurate for 2008. Without sufficient observation data, the predicted peak magnitude deviated far from its observed counterpart for the early weeks of each of the four years. As more observed data were fed into the model, the ensemble of variables and parameters were updated, and the peak magnitude accuracy continued to improve and approached 100% (i.e., zero offset). However, for 2008, as only five weeks of observation data were available for training the model, the forecast of the peak magnitude prediction was not comparable to the other three years. Notably, since the initial states for the ensembles and variables were set to establish the model, the forecast accuracy for the first week is not interpretable. Nevertheless, the time required for the peak magnitude prediction to converge is related to the initial states. Compared with 2009, the forecast accuracy of 2010 was high in the initial week, and the model only required five weeks to achieve a very accurate and stable prediction; conversely, nine weeks were required to achieve a similar level of accuracy for the forecasting of 2009.

**Figure 4:**
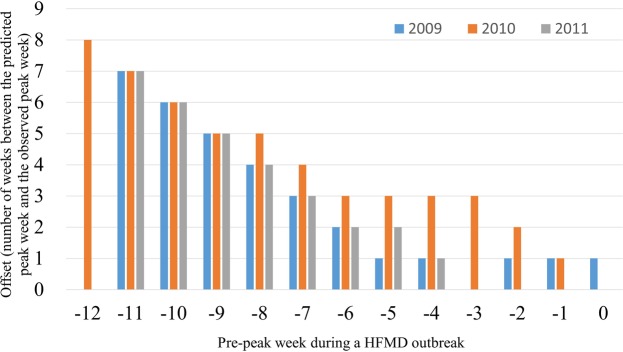
The forecast accuracy of the peak week arrival times for 2009, 2010, and 2011. The forecast result for 2008 is not included because of the lack of available training data before the peak week.

When evaluating the prediction accuracy of the peak week time, we excluded the year 2008 due to its shorter pre-peak data period. The accuracy of peak week forecasting was evaluated using the number of weeks of offset between the predicted peak week and the observed peak week (Fig. 4). As more observation data are used for peak week forecasting, the offset becomes smaller, showing a monotonic decreasing trend. For 2009, the offset was narrowed to one week as early as five weeks prior to the actual peak week. For 2011, the offset was narrowed to two weeks as early as six weeks before the actual peak week; additionally, no offset between the predicted peak week and the observed peak week was observed up to three weeks before the peak week. For 2010, the prediction offset was three weeks when forecast six weeks before the actual peak week, and it was within two weeks of offset when forecast two weeks before the actual peak.

**Figure 5:**
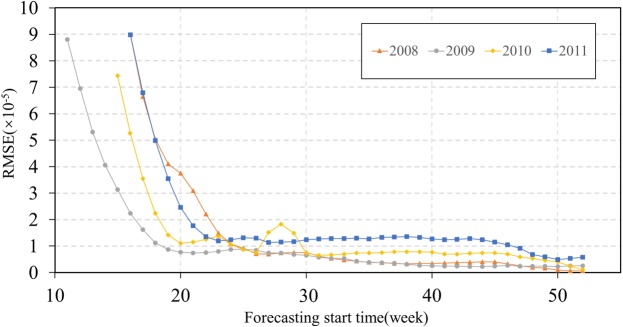
RMSE calculated for the forecast data and the observed data for 2008–2011.

The root-mean-square error (RMSE) between forecast data and observed data for the early weeks of forecasting appeared to be large and differed substantially across the four years (Fig. 5). These errors do not actually reflect the forecast quality because the forecast of the initial week was calculated using a randomly set initial state to run the SEIR- ensemble Kalman filter (EnKF) system. As the forecast was extended further into an outbreak season, more observation data were entered into the model, and the RMSE decreased sharply. Notably, the RMSE tended to remain low and relatively stable for all four years after a few weeks, suggesting that the performance of the model improves as more observation data are applied, thereby becoming more stable and reliable. This pattern also indicates that SEIR-EnKF forecasting is not very sensitive to the initial state, suggesting a minimum requirement of our forecasting model for additional or excessive data. Instead, as continuous observation data are entered into the forecasting system, the output converges to a reliable result. For 2009 and 2010, approximately 10 to 12 observation data points were needed before the RMSE decreased to a low and stable level. Importantly, 10–12 weeks is approximately the time frame for which data were available during these two years from the beginning of an outbreak to the peak week.

### Estimation of Transmission and Recovery Rates

**Figure 6:**
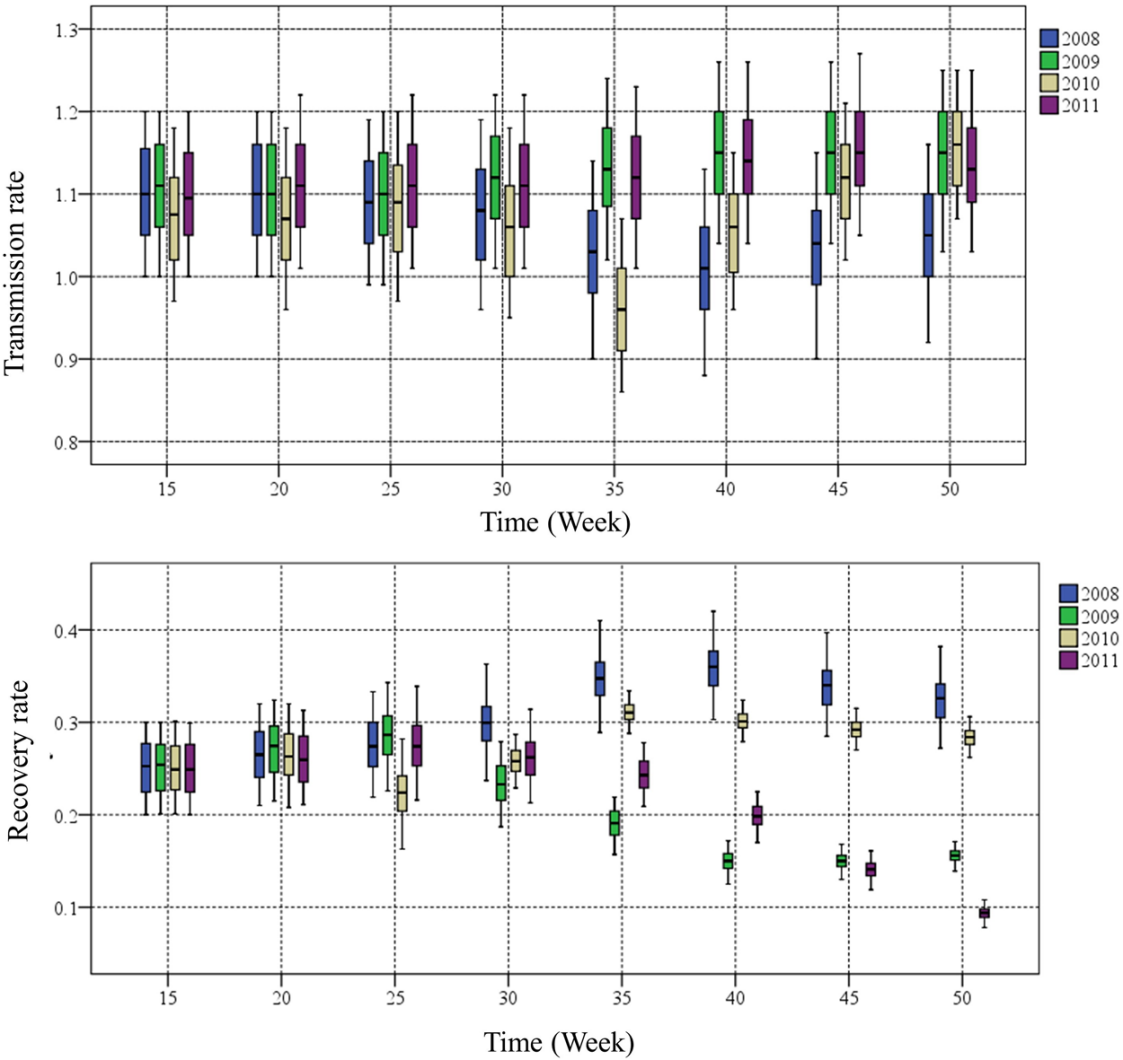
Estimation of the HFMD transmission and recovery rates during the indicated weeks in each of the four years using the SEIR-EnKF model. The thick horizontal line is the median, the edges of the boxes are the 25^th^ and 75^th^ percentiles, and the whiskers span the full range.

Estimates of the transmission and recovery rates for selected weeks using SEIR-EnKF are illustrated in Fig. 6. The transmission rate showed different trends across the four years. The transmission rates for 2009 and 2011 showed a monotonic increasing trend. However, in 2008 and 2010, the transmission rates first decreased until they reached their lowest point at approximately week 35, after which the rates increased. The recovery rates remained at the same level between the 15^th^ and 20^th^ weeks, after which they showed slight increasing trends in 2008 and 2010 and clear decreasing trends in 2009 and 2011. In summary, the HFMD transmission and recovery rates in 2008 and 2010 shared similar patterns that were clearly different from the patterns observed in 2009 and 2011.

The two distinct patterns of the HFMD transmission and recovery rates during 2008–11 may be related to multiple factors. First, the estimates of the transmission and recovery rates are related to the initial settings of the variables. Second, disease transmission and recovery rates are closely related to the social and environmental context of an outbreak, including factors such as weather conditions and the implementation of disease control measures. For HFMD outbreaks in Beijing, the dominant virus shifts between EV-71 and CV-A16. As shown in Supplementary Fig. 3, EV-71 was the dominant virus in 2008 and 2010, whereas in 2009 and 2011, it was CV-A16. Viruses may respond differently to changes in climatic factors, leading to the different patterns in the transmission and recovery rates observed across the four outbreak seasons investigated in this study.

## Discussion

HFMD is a high-risk childhood disease for East Asian countries, but its prediction is difficult due to complex influencing factors. In this study, we developed a real-time forecasting model for HFMD outbreaks by integrating SEIR and EnKF. The integrated SEIR-EnKF forecasting system assimilated real-time observation data into forecasts in a dynamic manner and exhibited good performance for the real-time forecasting of HFMD by predicting the peak time and magnitude with acceptable accuracy.

Based on empirical testing, the SEIR-EnKF system is reliable for real-time forecasting of HFMD. In 2008, the lack of pre-peak observation data for assimilation resulted in a relatively weak forecast. The forecasts of the magnitude of the infection rate and the time of the first peak were unclear. From 2009 to 2011, the system performed well in forecasting both the peak week timing and peak magnitude. The forecast accuracy was also assessed using RMSE, which showed a decreasing trend over time. Therefore, the integrated SEIR-EnKF system delivered real-time forecasts with short time intervals (e.g., weekly), and the forecast continuously improved as more observation data became available for assimilation. Our SEIR-EnKF forecasting system is a promising tool that may serve as an essential component in a warning system to assist public health agencies and the public in disease management and control measures in response to an HFMD epidemic.

Transmission and recovery rates are essential parameters for understanding an infectious disease. To a large extent, their values determine the status of an on-going epidemic. The size of the population infected with HFMD is largely decided by the transmission rate^21^. Compared with incubation and recovery rates, transmission dynamics are affected by many factors, such as geolocation, climate, the activities of susceptible populations, and government control measures. Therefore, it has the greatest uncertainty in the model, and the basic reproductive number^35^, which is defined as the transmission rate divided by the recovery rate, reflects whether an outbreak is spreading or controlled. However, a few previous studies have discussed transmission dynamics in detail. In 2008 and 2010, the transmission rate initially decreased and then increased throughout the year, whereas the transmission rate exhibited relatively small change during the seasons in 2009 and 2011. The recovery rates for 2008 and 2010 increased, whereas the recovery rate exhibited a continuous decrease throughout 2009 and 2011. The four-year estimated parameters provide a model-level explanation for the dynamic changes in the infection rate. It is showed that the combination of changes in the transmission rate and recovery rate contribute to the trend in the infection rate. According to the available four-year data analysed in our study, the different patterns in the years investigated might be associated with the virus type and corresponding features. Thus, the discovered patterns may be helpful for preparing a more accurate forecast. However, the changes in parameters at each step are difficult to explain due to the contributions of multiple, complex factors mentioned by other studies, such as control strategies 22 and cultural practices 33. In fact, these factors are uncertain and not easy to study quantitatively. Continuous monitoring of the parameters and the use of observed data will help establish a more accurate forecast in the future.

Our study has several limitations. First, the speed of forecast error convergence is not sufficient, as it decreased to a low level after the peak time in our study. This parameter might be improved by employing other assimilation technologies, such as the Particle filter. Second, we did not accurately predict the second peak in this study. Although the second peak in 2008 was forecast, its accuracy was not satisfying. In addition, we only used limited observation data to assimilate all parameters in the model, which may increase model uncertainty. Although we adjusted for this uncertainty by discussing the initial parameters and prior information of model parameters, more types of observed data would be better. These data directly related to HFMD, such as search engine data and weather data, would be helpful for the forecast and improve the speed of forecast error convergence.

In our study, we simplified the discussion of the possible association between virus types and transmission patterns. Further research is warranted to obtain a better understanding of this association. Long-term data for virus types and infection rates will be obtained and analysed using the SEIR-EnKF to discover and quantitatively describe the deterministic association. Once the connections are clearly identified, virus types will be incorporated into the SEIR-EnKF system for more accurate predictions. In addition, other factors, such as population structure, public health literacy, and weather conditions, may also impact the outbreak patterns. Future investigations should seek to determine the quantitative descriptive relationships with HFMD and incorporate these factors into the SEIR-EnKF system as well for more accurate forecasting of HFMD outbreaks.

## Methods

Ethics statement. This study was based on HFMD data in Beijing. All records were anonymized and no individual information can be identified. The research study protocol was approved by the Institutional Review Board at the Beijing CDC. All methods were performed in accordance with the principles of the Declaration of Helsinki.

### SEIR Model

The SEIR model is a dynamic model that considers the incubation period of a disease. It considers four groups of people: the susceptible (S), the exposed (E), the infected (I) and the recovered (R). The model is expressed using the following equations:

1 $$\frac{dS(t)}{dt}=-\beta (t)\frac{S(t)I(t)}{N}$$

2 $$\frac{dE(t)}{dt}=\beta (t)\frac{S(t)I(t)}{N}-\sigma (t)E(t)$$

3 $$\frac{dI(t)}{dt}=\sigma (t)E(t)-\gamma (t)I(t)$$

4 $$\frac{dR(t)}{dt}=\gamma (t)I(t)$$

5 $$N(t)=S(t)+E(t)+I(t)+R(t)$$

where t denotes time, *S(t)* is the number of susceptible people in the overall population at time t, *E(t)* is the number of exposed people, *I(t)* is the number of infected people, and *R(t)* is the number of recovered people who are no longer included in the transmission cycle. *β*(t) is the transmission rate from infectious people at time *t*, σ(t) is the incubation rate at time *t*, which is the inverse of the incubation period, *γ*(*t*) is the case recovery rate at time *t*, and *N* is the population size. The model does not consider deceased people because the death rate in the data used in this study was extremely low compared with the infection rate.

### EnKF Method

EnKF uses an ensemble to calculate error covariance based on stochastic theories. The median of ensemble states is assumed to be an optimal estimate of the true state. The forecast generates the prediction at time *k* + 1 based on data analysed at time *k* using a mechanical model, SEIR in this study. This step is often performed using a dynamic model. The following formula is used:

6 $${X}_{k+1}^{f}={M}_{k}{X}_{k}^{a}$$

where ${X}_{k}^{a}$ is the status at time *k* and ${X}_{k+1}^{f}$ is the forecast at time *k* + 1. *M*_*k*_ is the model system (SEIR model in this study) at time *k*.

A set of forecasts are generated, and the error terms of the forecasts are analysed as follows:

7 $${P}_{k+1}^{f}=\frac{1}{N-1}\sum _{i=1}^{N}\,({X}_{i,k+1}^{f}-\overline{{X}_{k+1}^{f}}){({X}_{i,k+1}^{f}-\overline{{X}_{k+1}^{f}})}^{T}$$

8 $${P}_{k+1}^{f}{H}^{T}=\frac{1}{N-1}\sum _{i=1}^{N}({X}_{i,k+1}^{f}-\overline{{X}_{k+1}^{f}}){(H{X}_{i,k+1}^{f}-H\overline{{X}_{k+1}^{f}})}^{T}$$

where *H* is the observation operator. The observation operator is a matrix which plays a role in linking the model variable vector and observations in the assimilation system (EnKF in this study). After converting the model variable vector to the form of observations by the observation operator, the variable vector and observed data could be in the same form for further analysis. N is the size of ensemble, ${X}_{i,k+1}^{f}$ is the variable vector at time *k* + 1, $\overline{{X}_{k+1}^{f}}$ is the mean of the variables vectors at time *k* + 1, and ${P}_{k+1}^{f}\,$is state error. The results obtained from Eqs (7) and (8) are used to calculate the Kalman gain matrix, as denoted by K_k+1_ (see Eq. 9 below), which is further used for balancing observation data and modelling results.

9 $${K}_{k+1}={P}_{k+1}^{f}{H}^{T}{(H{P}_{k+1}^{f}{H}^{T}+R)}^{-1}$$

where *R* is the error covariance of the observation. Finally, *K* is used to forecast the variables at the next time point:

10 $${X}_{i,k+1}^{a}={X}_{i,k+1}^{f}+{K}_{k+1}({z}_{k+1}-H{X}_{i,k+1}^{f})$$

where *z*_*k*+1_ is the observation data at time *k* + 1. We used the results of this analysis as the updated variable to forecast the model parameters and variables at the next time point.

### Initiation of the SEIR-EnKF Framework

The beginning week of an outbreak season is defined as the first week when the HFMD weekly infection rate reaches 2e-06, and it increases at a rate of 50% or more per week. The incubation period is generally 3–7 days^36^. The incubation period of HFMD among schoolchildren increases as they age^37^, and the mean incubation period for kindergarten students who are approximately 2–5 years of age is 4.4 days. In our data, the majority of the infected population was children younger than 5 years, corresponding to an incubation rate of (0.2, 0.3). Approximately 7 days are generally required to recover from HFMD^38^. The recovery period of HFMD caused by EV-71 is 4–6 days^39^ and 5–7 days for HFMD caused by a CV-A16 infection^40^. Therefore, we concluded that (0.1, 0.4) is a meaningful range for the recovery rate from HFMD compared with the incubation and recovery rates.

In addition, we defined the initial susceptible population *S(0)* to range from (0.8 N, N), where N is the proportion of the susceptible population, and the initial exposed population *E(0)* was in the range (5e-4N ± 10%). The range of the initial infected population *I(0)* was calculated by adding a 10% disturbance based on the observed infected population.

**Figure 7:**
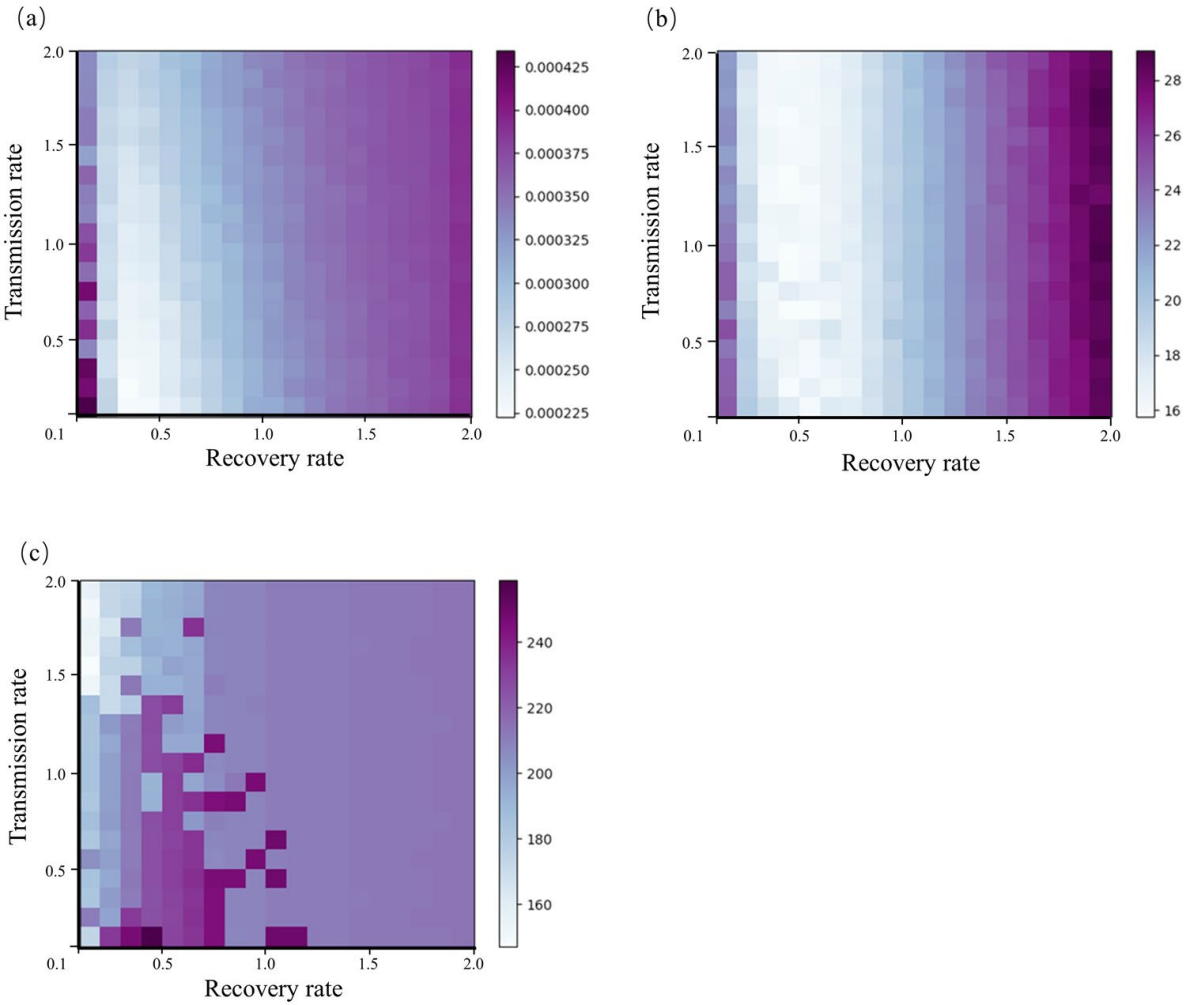
The cumulative error of (**a**) the infection forecasting, (**b**) the peak magnitude forecasting, and (**c**) the peak time (week) forecasting. These errors are defined as $\sum _{i=1}^{n}\sum _{j=1}^{m}{e}_{ij}$, $\sum _{i=1}^{n}\sum _{j=1}^{m}|FP{T}_{ij}-TP{T}_{i}|$ and $\sum _{i=1}^{n}\sum _{j=1}^{m}|FP{M}_{ij}-TP{M}_{i}|$ respectively. In these equations, n is the number of years and m is the number of steps in the forecast. *e*_*ij*_ is the RMSE between forecast infection rate of *j*_*th*_ step in *i*_*th*_ year and the observed data; *FPT*_*ij*_ is the forecast peak time of *j*_*th*_ step in *i*_*th*_ year; *TPT*_*i*_ is the true peak time in *i*_*th*_ year, *FPM*_*ij*_ is the forecast peak magnitude of *j*_*th*_ step in *i*_*th*_ year, and *TPM*_*i*_ is the true peak magnitude in *i*_*th*_ year.

We conducted a series of parameter sensitivity analyses to assist in the selection of the initial parameters for the transmission and recovery rates in the HFMD forecast (Fig. 7). We calculated the model error by running the SEIR-EnKF framework with different combinations of transmission and recovery rates. The transmission and recovery rates ranged between 0.1 and 2.0, and each parameter value was divided into 19 equal-length segments. As shown in Fig. 7(a), the cumulative error of the infection rate was high when the recovery rate was (0.1, 0.2); the error decreased as the recovery rate increased to 0.5, beyond which the error and recovery rate showed a positive relationship. However, model errors are generally not sensitive to changes in the transmission rate. Overall, when the recovery rate was (0.2, 0.6), and the transmission rate was (0.1, 1.5), the lowest error of the infection rate was observed. The error of the peak magnitude forecast was low when the recovery rate ranged from 0.2 to 0.8, and the error was not sensitive to the transmission rate (Fig. 7(b)). In Fig. 7(c), when the recovery rate was (0.1, 0.3), the cumulative error of the peak time was relatively low; but as the recovery rate increased from 0.3 to 0.8 and the transmission rate was less than 1.5, the error generally remained high. Therefore, we set the initial transmission rate to (1.0, 1.2) and the initial recovery rate to (0.2, 0.3) to keep the three aforementioned types of error low in our forecasting model.

## Supplementary information

Real-Time Forecasting of Hand-Foot-and-Mouth Disease Outbreak through Integrating Compartment Model and Assimilation Filtering

## Data Availability

We obtained data from 72,266 HFMD cases reported in Beijing during 2008–2011 from the Beijing Center for Disease Control and Prevention. For each case, the incidence date and diagnosis date were recorded. The number of individuals in the overall population and children in Beijing were obtained from the Beijing Bureau of Statistics.
