# Supplementary material for: Real-Time Forecasting of Hand-Foot-and-Mouth Disease Outbreaks using the Integrating Compartment Model and Assimilation Filtering
Source: Sci Rep. 2019 Feb 25;9:2661. doi: 10.1038/s41598-019-38930-y (PMC6389963; doi:10.1038/s41598-019-38930-y)
Supplement: Supplementary file 1 — Real-Time Forecasting of Hand-Foot-and-Mouth Disease Outbreak through Integrating Compartment Model and Assimilation Filtering [file 41598_2019_38930_MOESM1_ESM.docx]

**Title:** **Real-Time Forecasting of Hand-Foot-and-Mouth Disease Outbreak using the Integrating Compartment Model and** **Assimilation Filtering**

**Zhicheng Zhan^1^, Weihua Dong^1,*^, Yongmei Lu^2^,** **Peng Yang^3^, Quanyi Wang^3^, Peng Jia^4,5^**

## Comparison between prior forecast and posterior analysis

Prior forecast and posterior analysis are two key steps for HFMD forecast. The performance of prior forecast only decided by prior step of model parameters, the posterior analysis step combine the prior forecast result and the current observed data to generate more credible data. It is indicated that posterior analysis fit the observed data better from supplementary Fig. 1


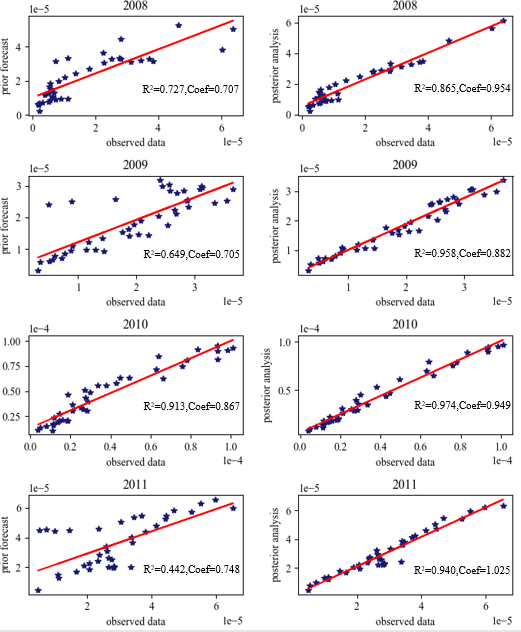


**Supplementary Fig. 1** Correlation between the observation data and the prior forecast (left panel) and between the observation data and the posterior analysis (right panel). Coef (coefficient) and$R^{2}$ of fit between observation data and posterior is given. $R^{2}$ between the observation data and the posterior analysis is larger than that between the observation data and prior forecast.

## Description of Real-time Forecasting of HFMD

We performed real-time forecasting of HFMD for 2008-2011. Supplementary Figure S2 shows the ensemble forecast and the median of the ensemble forecast of infection rate $I_{r}$ based on observation data for the selected weeks during each of the four years. For the first few weeks and with limited observation data, the forecast outputs did not match the observation very well. However, as more observation data were entered into the forecasting system for the later weeks, the forecast outputs became more accurate, a consistent trend through the weekly forecasts for each of the four years, as illustrated in Figure S2. Specifically, after a parameter optimization period during which observed data are continuously fed into the model to calibrate the parameters, our SEIR-ENKF model prediction becomes stable and converges. In 2008, the first and second peaks appeared in the 20^th^ and 27^th^ weeks. Before running the forecast model for week 23, the system only predicted the first peak. Starting from week 23, the system consistently forecasted the second peak of HFMD, giving a four-week window before the onset of the second peak. For the years of 2009, 2010, and 2012, the forecasts of the peak week and peak $I_{r}$ continued to improve as the model approached the real peak week each year. Notably, with the peak week data added to the model, the forecast for post-peak weeks continued to improve.

**
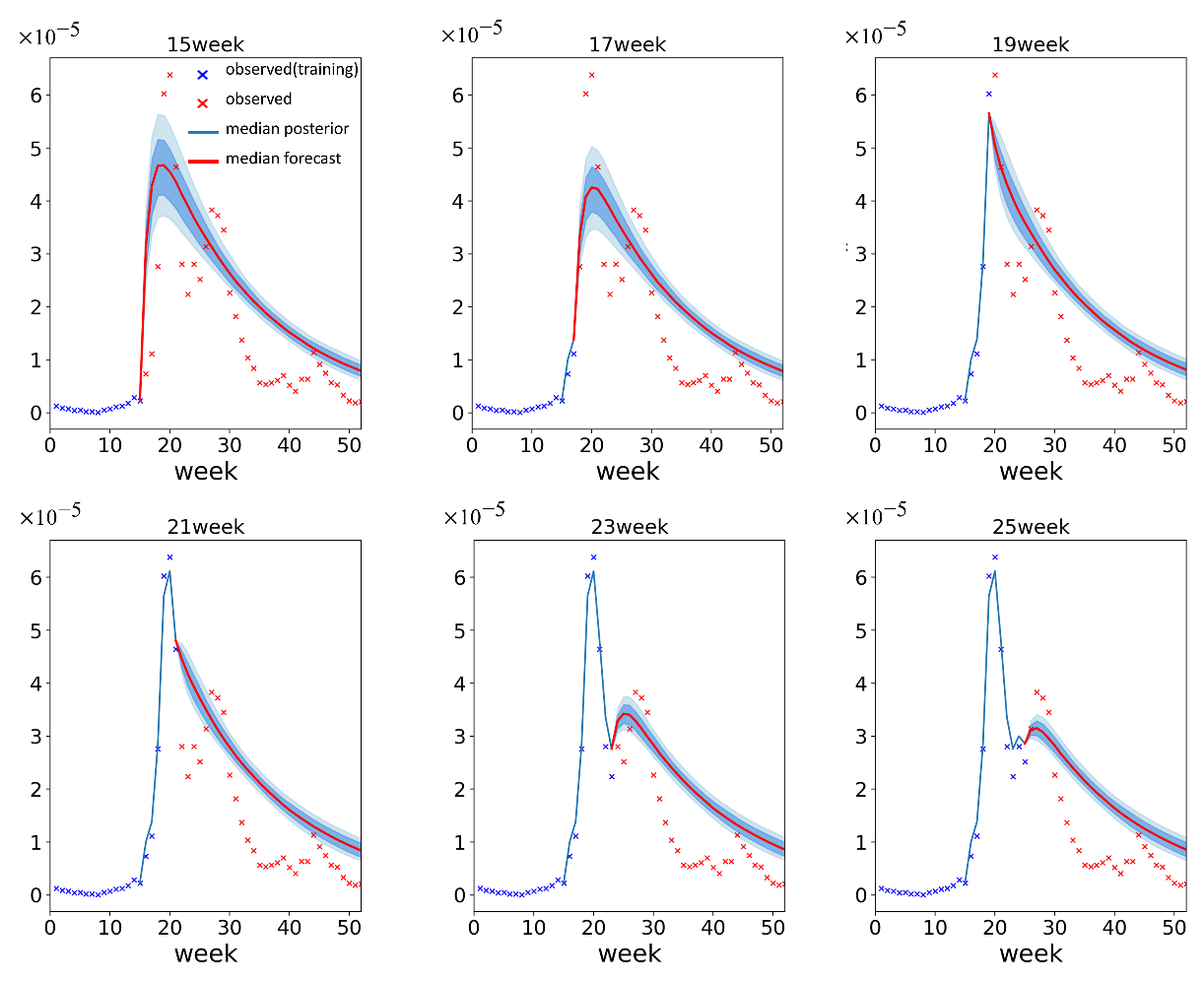
**

1. 2008

**
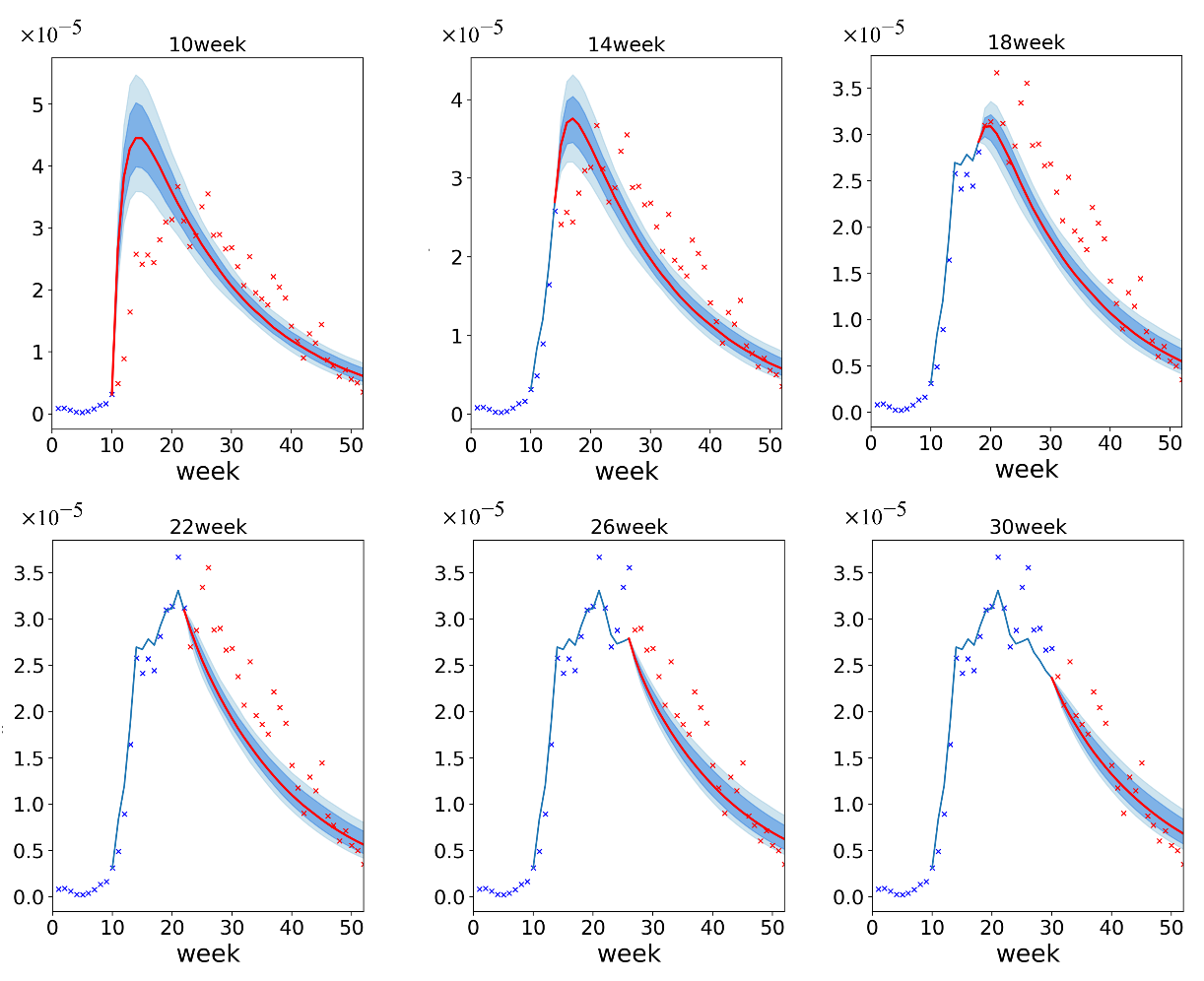
**

1. 2009

**
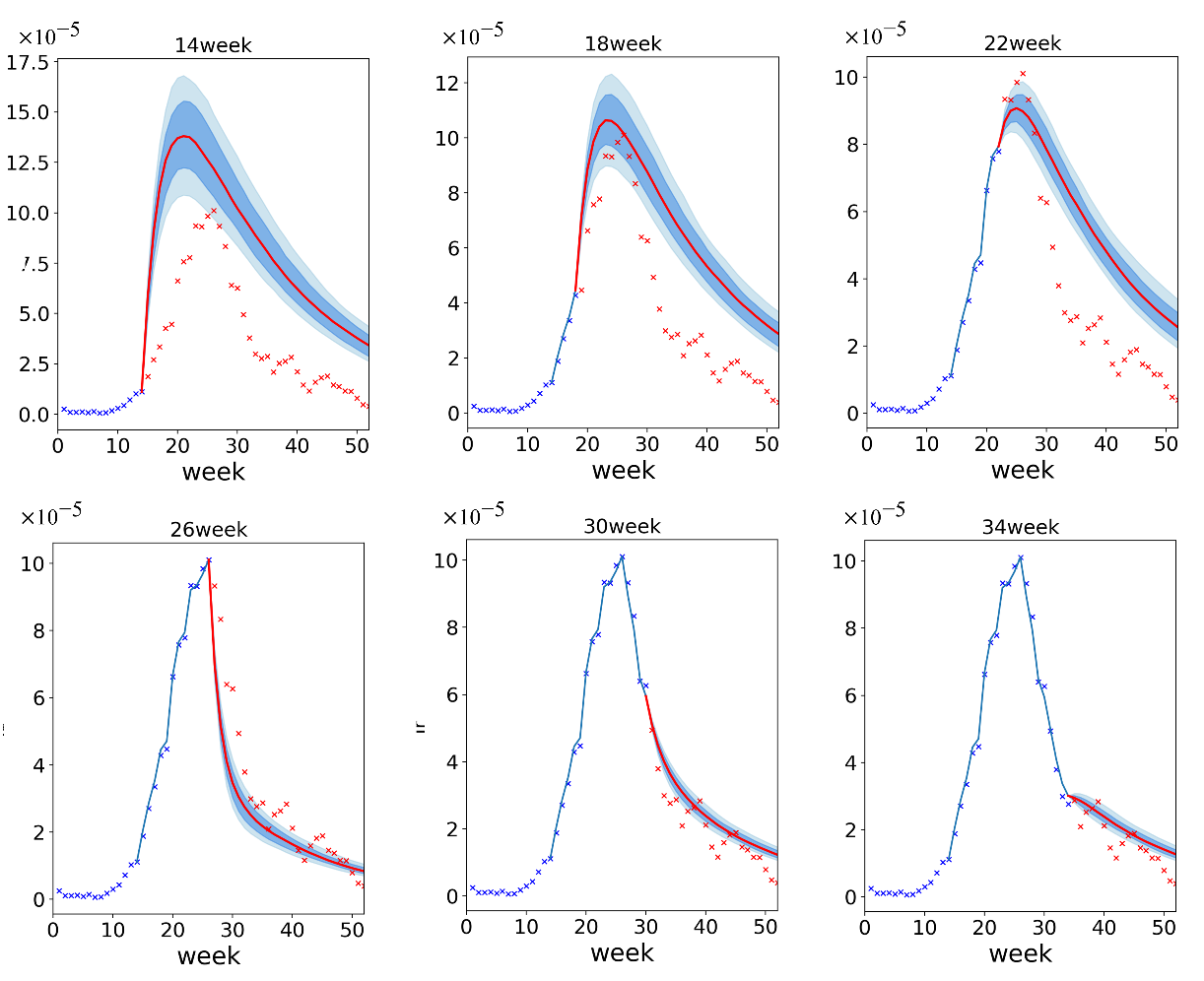
**

(c) 2010


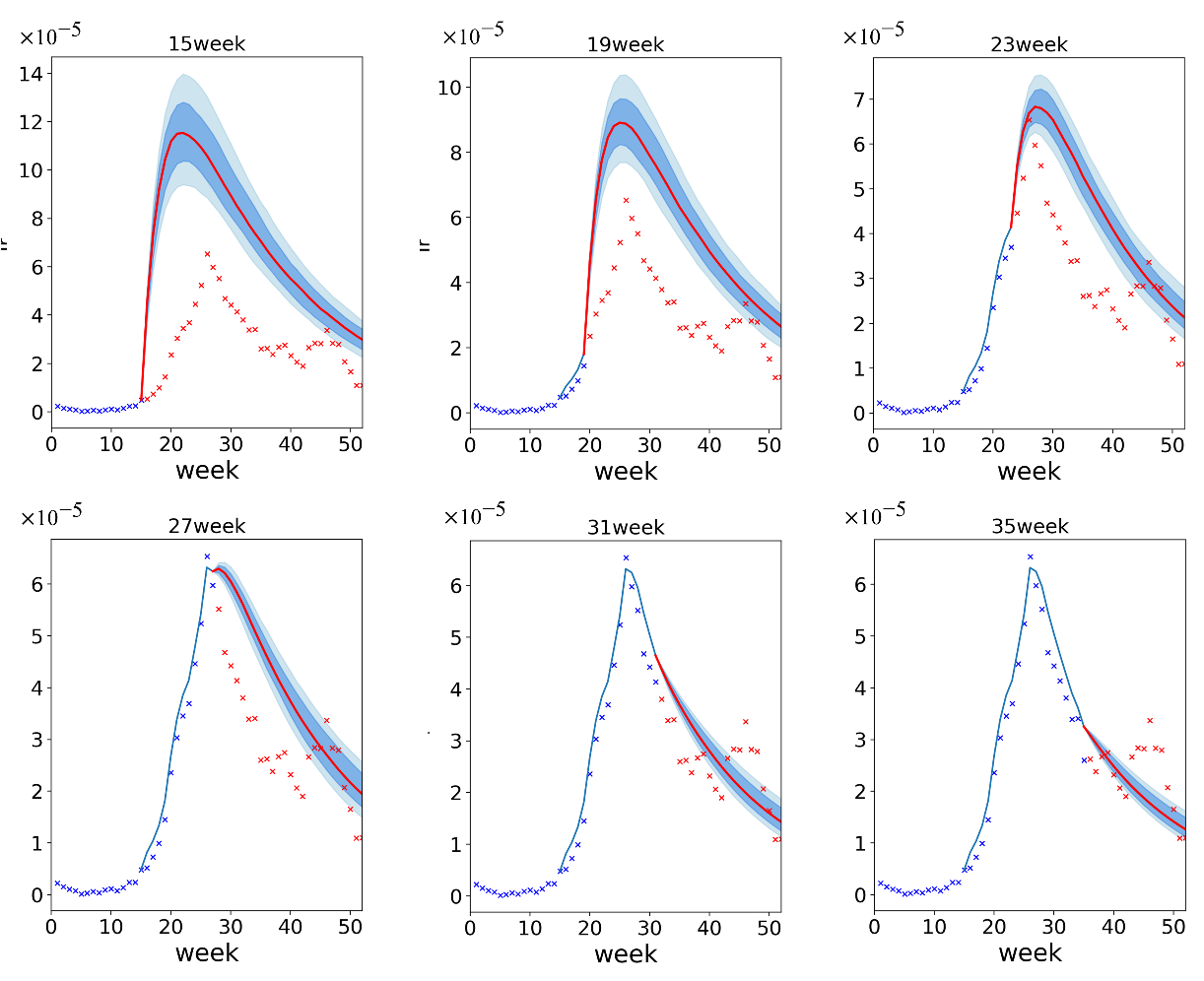


(d) 2011

**Supplementary Fig. 2** Real-time forecasting of the infection rates for the selected weeks: 2008(a), 2009(b), 2010(c), and 2011(d). The blue dots represent observation training data up to a selected week of forecasting; the data are used to adjust the model parameters and variables. The red dots represent the observation data after a selected week; the data are used to evaluate the forecasting model. The light blue lines represent the model prediction of the infection rate up to a selected week. The red lines represent the median of forecasts for the infection rate following a selected week; a forecast is generated based on data for each week up to a selected week. The blue shading shows the distribution of the ensemble at 10% and 25%.

## Virus types that cause HFMD

Many types of virus can cause HFMD, but EV71 and CV-A16 are dominant, and it is noticeable that EV71 caused more cases than CV-A16 in 2008 and 2010 while in 2009 and 2011,CV-A16 become the virus that caused the most cases. Other types of virus caused case percentage no more than 25% during 2008-2011. All data are laboratory confirmed cases.


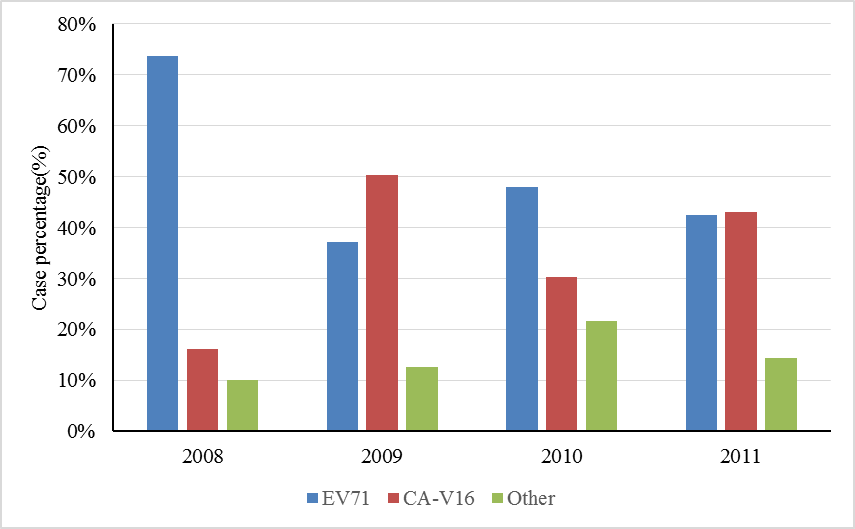


**Supplementary Fig. 3** Percentages of HFMD cases that can be attributed to EV71, CV-A16, and other enteroviruses.
